# Fatal drug reaction to andexanet alfa: a case report

**DOI:** 10.1002/jha2.959

**Published:** 2024-06-22

**Authors:** Richard J. Buka, Mamidipudi T. Krishna, David J. Sutton

**Affiliations:** ^1^ Institute of Cardiovascular Sciences University of Birmingham Birmingham UK; ^2^ Institute of Immunology and Immunotherapy University of Birmingham Birmingham UK; ^3^ Clinical Haematology University Hospitals North Midlands NHS Trust Stoke‐on‐Trent UK; ^4^ Clinical Haematology Mid Cheshire Hospitals NHS Foundation Trust Crewe UK

**Keywords:** anaphylaxis, andexanet alfa, case report, drug reaction, thrombosis

## Abstract

Andexanet alfa is a recombinant, modified factor Xa (FXa) molecule that is used for the reversal of the anticoagulant effect of oral anti‐FXa anticoagulants in patients with major haemorrhage. Here, we present a case of an 85‐year‐old man taking rivaroxaban for atrial fibrillation, who presented with an acute, upper gastrointestinal bleed. He was stabilised with red cell transfusion and then received a 400 mg bolus of andexanet alfa. Within minutes of this, he developed chest tightness, shortness of breath, ischaemic electrocardiographic changes and then cardiac arrest from which he could not be resuscitated. The onset of symptoms was clearly temporally related to andexanet alfa administration and the differential diagnosis includes anaphylaxis with Kounis syndrome, or myocardial infarction. Although infusion site reactions have been reported and are relatively common, this is to date the first case of a fatal drug reaction andexanet alfa. This knowledge can be factored into physicians’ risk–benefit decisions when treating patients with oral anti‐FXa anticoagulant‐associated major haemorrhage.

## 1 INTRODUCTION

Andexanet alfa is a modified factor Xa (FXa) molecule that has a conditional license for the reversal of oral anti‐FXa anticoagulants. Although a randomised trial of patients with intracerebral haemorrhage (ANNEXA‐I) showed decreased haematoma expansion in treated patients, benefits in survival, disability or quality of life have not been proven [1]. For gastrointestinal haemorrhage, there is only a single‐arm trial (ANNEXA‐4) thus efficacy is again, not proven [2]. Furthermore, there is concern that andexanet alfa is associated with a high risk of arterial thrombosis [1, 2]. Despite this, andexanet alfa is fairly well tolerated, with one serious infusion reaction occurring in ANNEXA‐4 [2]. In healthy volunteers, mild infusion‐related reactions occurred in 39/223 (18%) participants with symptoms such as flushing [3]. To our knowledge, no cases of anaphylaxis have been reported.

Here, we report the first case of a fatal drug reaction after andexanet alfa administration.

## 2 CASE

In September 2023, an 85‐year‐old man was admitted to hospital following a fall at home. He had a past medical history of atrial fibrillation, stroke, previous excess alcohol intake, osteoarthritis and falls. Prior to admission, he was independently mobile at home with the aid of walking sticks. His on‐going medications included rivaroxaban 20 mg once daily, thiamine 100 mg once daily, salbutamol inhaler as required and paracetamol as required. He had been taking rivaroxaban since 2018. There were no known allergies or history of anaphylaxis. On examination, he had infected‐looking, chronic leg ulcers but no rash was reported. His weight was 98 kg. His inflammatory markers were raised, and he was treated with antibiotics and supportive therapy. A wound swab grew group B beta haemolytic streptococcus.

Despite gradual improvement in his clinical condition and inflammatory markers (Table 1), on the 16th day of his admission (30/9/2023), he became tachycardic, hypotensive and developed melaena. At 20:55, he was reviewed urgently as his blood pressure had dropped from 139/66 earlier in the day to 93/46. His heart rate had increased to 92 beats per min (bpm) from 78 earlier in the day. Rivaroxaban had last been given in the morning at 8 am, over 12 h prior. Urgent laboratory tests showed a haemoglobin of 58 g/L and urea of 13.1 mmol/L consistent with an upper gastrointestinal bleed. Creatinine was 101 µmol/L and international normalised ratio (INR) was 1.4. Laboratory results over the admission are summarised in Table 1; a gradual decline in haemoglobin is noted.

**TABLE 1:** Selected laboratory results from admission to death.

Parameter	15/9/23	18/9/23	21/9/23	23/9/23	25/9/23	26/9/23	29/9/23	30/9/23 20:45
Haemoglobin (g/L)	128	121	111	104	96	91	88	56
Platelets (×10^9^/L	270	225	300	355	415	428	441	397
WCC (×10^9^/L)	20.3	21.2	33.7	18.2	15.0	12.3	9.4	11.1
CRP (mg/L)	156	323	235	105	73	69	46	48
Creatinine (µmol/L)	113	109	109	90	87	87	87	101
Urea (mmol/L)	5.7	8.4	10.5	8.3	5.0	4.5	4.2	13.1
eGFR	51	53	53	67	70	70	70	58
INR	1.7	1.2	NP	NP	NP	NP	NP	1.4

Abbreviations: CRP, C‐reactive protein; eGFR, estimated glomerular filtration ratio; INR, international normalised ratio; NP, not performed; WCC, white cell count.

Fluids, red cell transfusion and omeprazole infusion were commenced. Red cell transfusion was started at 22:45 and completed at 01:45. Following this, the patient was stable; blood pressure was 107/51 and heart rate was 87 bpm.

After consultation with a haematologist and a gastroenterologist, it was agreed that andexanet alfa should be given and oesophagogastroduodenoscopy (OGD) performed in the morning. An intravenous bolus dose of andexanet alfa 400 mg was given at approximately 02:15. Soon afterwards, he became acutely unwell and at medical review at 02:40, it was documented that he was complaining of shortness of breath and chest tightness. The andexanet alfa infusion was not started. On examination he was pale, mottled and diaphoretic. Electrocardiogram (ECG) showed ST depression and T wave inversion in the lateral leads. At 02:50, he became unresponsive with no cardiac output. Cardiopulmonary resuscitation was started but spontaneous circulation could not be restored, and the patient died.

## 3 DISCUSSION

The temporal relationship between the administration of andexanet alfa, the onset of symptoms and cardiac arrest provides compelling evidence for a fatal drug reaction. This is therefore, to our knowledge, the first reported case of a fatal drug reaction to andexanet alfa.

It is our view that the most likely diagnosis is anaphylaxis, which is defined by the World Allergy Organization as ‘*…a serious systemic hypersensitivity reaction that is usually rapid in onset and may cause death’* [4]. Evidence against anaphylaxis as the cause of death is that there were no skin or mucosal symptoms reported, and that the final vital sign measurements prior to cardiac arrest showed hypertension rather than hypotension. Still, in anaphylaxis that occurs during anaesthesia, cutaneous symptoms are not present in over one quarter of patients whilst cardiovascular collapse occurs in half [5]. Unfortunately, given the rapid onset of symptoms, neither total serum total mast cell tryptase nor high sensitivity troponin levels were requested. Nevertheless, anaphylaxis is a clinical diagnosis [4] and serum total mast cell tryptase is a poorly sensitive test [6, 7, 8].

Features are also consistent with Kounis syndrome in which the release of inflammatory mediators in an allergic reaction leads to acute coronary syndrome occurring through coronary vasospasm or plaque rupture [9]. A further possibility is the presence of an underlying clonal mast cell disorder such as indolent systemic mastocytosis. This disorder commonly presents as cardiovascular anaphylaxis with a paucity of mucocutaneous signs [10, 11, 12]. In studies of andexanet alfa in healthy volunteers, the adverse drug reaction of flushing was seen in 6% [13], which raises the possibility of direct (non–IgE‐mediated) mast cell activation as can be seen with vancomycin and opiates. To our knowledge, this has not been investigated with andexanet alfa.

The modifications to FXa to create andexanet alfa may open‐up novel antigenic sites to which IgE antibodies may form. This patient had not previously been exposed to andexanet alfa but the presence of cross‐reacting antibodies that had previously formed to a similar molecule is possible. Another possibility is a reaction to excipients [14] but these ones that are commonly found in food and other medicinal products. Finally, the recombinant protein is produced in Chinese hamster ovary cells in serum‐free media and is purified by several steps, which include centrifugation, chromatographic purification, nanofiltration and virus inactivation [15]. Nevertheless, the presence of residual hamster proteins is likely and known allergic reaction to hamster proteins is listed as a contraindication by the European Medicines Agency [14].

Given the ischaemic changes on ECG, myocardial infarction as the primary cause of cardiac arrest is also possible. This could have occurred coincidentally, unrelated to andexanet alfa administration but again, the time course is highly suggestive of a causal effect of the drug. Andexanet alfa is associated with an excess risk of thrombosis, often arterial, several days following treatment [1, 2, 3]. This is attributed to the interaction between andexanet alfa and tissue factor pathway inhibitor, which is unperturbed by the modifications from FXa [16, 17]. However, such ultra‐acute induction of thrombosis has not been reported.

Clinicians may wish to take this report into account when designing policies and deciding on individual treatment decisions.

## AUTHOR CONTRIBUTIONS

Richard J. Buka wrote the manuscript, which was reviewed and amended by Mamidipudi T. Krishna and David J. Sutton.

## CONFLICTS OF INTEREST STATEMENT

Richard J. Buka is a named investigator on a grant from AstraZeneca investigating real‐world use of andexanet alfa in the United Kingdom. He has received honoraria from Bayer, Sobi, Sanofi, Takeda and Viatris.

Mamidipudi T. Krishna received research funds from NIHR RfPB, MRC CiC, GCRF, FSA and University of Birmingham outside this work. His department at UHB has received educational grants for PracticAllergy course from ALK Abello, Allergy Therapeutics, MEDA, Thermo Fisher Scientific and other pharmaceutical companies over the years. He is Chair of Equality, Diversity and Inclusion working group of BSACI, co‐author for BSACI guidelines on penicillin allergy and an associate editor for Clinical Experimental Allergy.

David J. Sutton has received honoraria from Pfizer‐BMS and Bayer.

## ETHICS STATEMENT

This is a retrospective case report that had no bearing on patient care, and data are anonymised. As such, ethical approval was not required. The case was reported to the UK Medicines and Healthcare products Regulatory Agency via the Yellow Card reporting scheme for suspected adverse effects of medicines.

## PATIENT CONSENT STATEMENT

Consent for publication of this case report was given by the deceased patient's next of kin.

## CLINICAL TRIAL REGISTRATION

The authors have confirmed clinical trial registration is not needed for this submission.

## Data Availability

All data underlying the results are available as part of the article and no additional source data are required.
